# Knockdown a Water Channel Protein, Aquaporin-4, Induced Glioblastoma Cell Apoptosis

**DOI:** 10.1371/journal.pone.0066751

**Published:** 2013-08-12

**Authors:** Ting Ding, Ying Zhou, Kai Sun, Weizhong Jiang, Wenliang Li, Xiaoli Liu, Chunying Tian, Zhihui Li, Guoguang Ying, Li Fu, Feng Gu, Weidong Li, Yongjie Ma

**Affiliations:** 1 Tianjin Medical University Cancer Institute and Hospital, Key Laboratory of Breast Cancer Prevention and Therapy of the Ministry of Education; Key Laboratory of Cancer Prevention and Therapy of Tianjin, Tianjin, China; 2 Bio-X Institutes, Key Laboratory for the Genetics of Developmental and Neuropsychiatric Disorders, Shanghai Jiao Tong University, Shanghai, China; 3 Department of Urology, Renji Hospital, School of Medicine, Shanghai Jiao Tong University, Shanghai, China; 4 Department of Neurosurgery, the Fifth People's Hospital of Shanghai, Fudan University, Shanghai, China; University of Sydney, Australia

## Abstract

Glioblastomas are the most aggressive forms of primary brain tumors due to their tendency to invade surrounding healthy brain tissues, rendering them largely incurable. The water channel protein, Aquaporin-4 (AQP4) is a key molecule for maintaining water and ion homeostasis in the central nervous system and has recently been reported with cell survival except for its well-known function in brain edema. An increased AQP4 expression has been demonstrated in glioblastoma multiforme (GBM), suggesting it is also involved in malignant brain tumors. In this study, we show that siRNA-mediated down regulation of AQP4 induced glioblastoma cell apoptosis *in vitro* and *in vivo*. We further show that several apoptotic key proteins, Cytochrome C, Bcl-2 and Bad are involved in AQP4 signaling pathways. Our results indicate that AQP4 may serve as an anti-apoptosis target for therapy of glioblastoma.

## Introduction

Glioblastoma has a high proliferation ability and high tendency to invade diffusely into surrounding healthy brain tissues, thereby precluding their successful surgical removal [1], [2]. Aquaporins (AQPs) are a family of small hydrophobic, integral membrane proteins ranging from 26 to 34kDa in size. To date, 13 different AQPs (AQP 0–12) have been identified in mammals. These integral proteins have been found to form transmembrane water channels that play critical roles in controlling the water flow into and out of cells [3]. Both AQP1 and AQP4 have been clearly identified in the brain, and AQP4 is well known to participate mainly in brain edema [4]. AQP4 is primarily expressed at the border between brain parenchyma and major fluid compartments, including astrocyte foot processes, glia limitans, as well as ependymal cells and subependymal astrocytes [5]. This distribution suggests that AQP4 control water fluxes into and out of the brain parenchyma.

Apoptosis is an endogenous program of controlled cell death that has been implicated in many physiological and pathological processes. Since the incidental discovery of AQPs, they have been implicated in various diseases such as brain edema, cancer, nephrogenic diabetes insipidus, and were eventually associated with cellular apoptosis [6]. Morphologically, one of the earliest and highly conserved events in apoptosis is water loss and subsequent cell shrinkage. This process has recently been named the apoptotic volume decrease (AVD) [7]. A previous study showed that blocking AQP1 activity with HgCl_2_ prevented AVD and the subsequent downstream apoptotic events such as cell shrinkage, DNA degradation, loss of mitochondrial membrane permeability and caspase 3 activation [8]. Kong et al have indicated that AQP4 deficiency inhibited the proliferation, survival, migration and neuronal differentiation of adult neural stem cells derived from the subventricular zone of adult mice [9]. Our previous study also showed that reduction of AQP4 induced impaired migration and invasion of human glioma cells [10]. Although AQP4 has been implicated in AVD, the exact mechanisms that trigger the changes in the cellular volume remain to be elucidated [11].

In this study, we tested the hypothesis that AQP4 directly participates in glioblastoma cell proliferation and apoptosis. We show that down-regulation of AQP4 using a specific siRNA or an inhibitor, phorbol 12-myristate 13-acetate (PMA), induced apoptosis and impaired the proliferation of the LN229 and U87 Human glioblastoma cell lines. Further, our data demonstrate that several key proteins, cytochrome C, Bcl-2 and Bad which are critical for cell apoptosis are likely involved in AQP4 signaling pathways. Thus, our studies suggest that AQP4 is a critical regulator in glioblastoma cell apoptosis, and may serve as a therapeutic target for therapy of glioblastoma.

## Materials and Methods

### Cell culture and reagents

Human glioblastoma cell lines LN229 and U87 were obtained from American Type Culture Collection (Manassas, VA, USA) and were cultured in Roswell Park Memorial Institute (RPMI) 1640 medium containing with 10% Fetal Bovine Serum (FBS) (complete medium). The antibodies used were as follows: AQP4 (Chemicon AB3594) was purchased from Millipore (Billerica, MA, USA), and AQP1 (sc-20810), Cyt-C (sc-13156),Bad (sc-8044),Bcl-2 (sc-783), β-actin (sc-47778), and Ki67 (sc-15402) were all purchased from Santa Cruz Biotechnology, Inc (Santa Cruz, CA, USA). Neutral Red Staining Solution (0.1%) was from Santa Cruz Biotechnology, (Santa Cruz, CA).

### RNA interference

Cells were plated in a 35 mm dish for 24 h before transfection in the complete medium. The transfection was performed with Lipofectamine 2000 according to the manufacturer's instructions (Invitrogen, Carlsbad, CA). AQP4-specific siRNA plasmids for LN229 cells (insert: GCTCAATAGCTTTAGCAATTG and scrambled sequence inserted into pGPU6/GFP/Neo) were from GenePharma Corp. (Shanghai, China). To establish stable siAQP4 cell clones, the G418-resistant cells were screened and their expression level of AQP4 protein was monitored by Western blotting.

### Transient transfection of U87 cells with AQP4 siRNA

U87 cells were seeded into 6-well plates and 24 h later they were transiently transfected with 1 µM of either a control siRNA, or a human AQP4 siRNA oligos (5′-GCTCAATAGCTTTAGCAATTG-3′) and 28 µl of GenePorter Transfection Reagents (Gene Therapy Systems, San Diego. CA, USA). The transfected cells were cultured in complete medium for 36 h before experiments.

### Western blotting

Western blotting was performed as described by Zhang et al [12]. In brief, cells were lysed by 1×SDS lysis buffer (Tris-HCl, pH 6.8, 62.5 mM, 2% SDS, 10% glycerol) followed by centrifugation at 10,000 rpm for 10 min at 4°C. Equal amounts of cell lysates (30 µg total protein/lane) were loaded and separated by SDS-PAGE, and proteins were transferred onto nitrocellulose membranes. The membranes were probed with the primary antibodies as follows: anti-AQP4 (1∶1000), AQP1 (1∶1000), Cyt-C (1∶500), Bad (1∶1000), Bcl-2 (1∶1000) and β-actin (1∶5000). Alkaline phosphatase-conjugated secondary antibodies were then added, and immune complexes were detected with nitrotetrazolium. The results were visualized by using the mixture of Nitrotetrazolium blue chloride (NBT) and 5-bromo-4-chloro-3-indolylphosphate p-toluidine salt (BCIP). All of the western blotting results are shown in Figures S1 and S2.

### Osmotic Fragility Test

Cells were plated in 35-mm dishes at a density of 6×10^5^ cells/ml to form a monolayer. 24 h later, the medium was replaced with double-distilled water, and the cells were incubated for 2 min. Dead and living cells were stained using the Neutral red Staining Solution (0.1%) as follows. The dishes were washed twice with phosphate-buffered saline (PBS), and then incubated with Neutral Red Staining Solution (0.1%) at 37°C in 5% CO2 incubator for 30 min. Cells were fixed with 4% paraformaldehyde for 15 min. The living cells were counted under a light microscope at 200× [13].

### MTT assay

This assay detected the ability of viable cells to convert a soluble tetrazolium salt, 3-(4,5-dimethylthiazol-2-yl)-2,5-diphenyltetrazolium bromide (MTT), into a blue formazan end product by mitochondrial dehydrogenase enzymes. Briefly, cells (1000/well) were plated in 96-well plates in 100 µl of the complete medium. The medium was changed every two days. The details of MTT assay were in the following: Following overnight attachment, 10 µl of MTT stock solution (5 mg/ml of MTT in PBS) was added to each well. After incubation for 4 h at 37°C in 5% CO_2_ incubator, 150 µl dimethyl sulfoxide was added to each well and shook for 10 min, absorbance was then measured at 490 nm by a microplate reader. Triplicate wells with pre-determined cell numbers were subjected to the above assay concomitantly with the test samples to normalize the absorbance readings. The experiments were independently conducted in triplicate at least three times. MTT assays were performed for six consecutive days.

### Colonies formation assay

Type I collagen, 10×F-12 medium and reconstitution buffer (Cellmatrix Type CD, Nitta Gelatin) were mixed together on ice at a ratio of 8∶1∶1 respectively. The prepared tumor cell suspension was added into the collagen solution (1∶10, v∶v) at a final density of 3×10^5^ cells/ml. Three drops of the collagen-cell mixture (30 ul/drop) were placed in each well of a 6-well plate and allowed to gel at 37°C in a CO_2_ incubator; the final concentration was about 9×10^3^ cells per droplet. One hour later, 3 ml DMEM/F-12 medium containing 10% fetal bovine serum was added to each well and the plates were placed in an incubator at 37°C with 5% CO_2_ for ten days. Cells were fixed in 10% formalin and stained with Neutral Red Staining Solution (0.1%) at 37°C in 5% CO2 incubator for 30 min. The number of colonies was counted under a light microscope [13].

### Cytochrome C detection by flow cytometry

Cells were plated in 6 cm dish at 8×10^5^ cells per dish and cultured in a complete medium at 37°C in 5% CO_2,_ and 24 h later cells were washed 3 times with cold PBS, and then were digested and centrifuged at 1000 rpm for 5 min, cell were then suspended in 500 µl of PBS. After that, the cells were incubated with TIRTC anti-Cytochrome C antibody at room temperature for 60 min in the dark and Cytochrome C efflux from mitochondria was detected by flow cytometry.

### DAPI staining

Cells were plated in 24-well plates at 5×10^4^ cells per well and cultured in a complete medium at 37°C in 5% CO_2_, 24 h later cells were washed with PBS and fixed with 4% paraformaldehyde for 20 min at room temperature, then cells were treated with 0.1% TritonX-100 for 10 min on ice and cells were stained with 1 µg/ml DAPI to visualize the nuclei. The images were acquired using a fluorescent microscope at a magnification of 200×.

### Apoptosis detection

An annexin V-R-PE apoptosis detection kit was applied to determine the cell apoptosis by flow cytometry. In preparation, cells were washed with ice cold PBS and were re-suspended in 100 µl of binding buffer, and stained with 10 µl of PE-conjugated Annexin-V and 10 µl of 7-AAD. The cells were incubated for 15 min at room temperature in the dark, and then 400 µl of binding buffer was added for detection by flow cytometry.

### Ethics Statement and Tumorigenicity Assay

Male athymic Nu/Nu mice (4 to 5 weeks-of age) were purchased from Wei Tong Li Hua Experimental Animal Co. Ltd. (Beijing, China). All animals received humane care according to the criteria outlined in the Guide for the Care and Use of Laboratory Animals prepared by the National Academy of Sciences and published by the National Institutes of Health. All protocols were approved by the Tianjin Medical University Cancer Institute & Hospital IACUC (Institutional Animal Care and Use Committee) and Ethics Committee of the Animal Facility of Tianjin Medical University Cancer Institute & Hospital. All efforts were made to minimize suffering. A total of 3×10^6^ cells were subcutaneously inoculated into one flank of each mouse. Tumor size was measured each week and the mice were sacrificed after six weeks. The tumors were dissected and their size was measured using a digital caliper. The experimental and control groups each comprised 24 animals. The volume (V) of the tumors was obtained by using the following equation: V = 1/2 ab^2^, where a and b is the larger and smaller diameter of the subcutaneous tumor [14].

### Immunohistochemistry

Immunohistochemisty was performed using standard techniques. Transplantation tumor tissues were obtained from subcutaneously inoculated Nu/Nu mice. Antigen retrieval was performed by autoclaving. Incubation with 10% serum in PBS was performed for 15 min to eliminate nonspecific staining. Incubation with primary antibody was carried out over night. After washing unbound antibody, sections were treated with the horseradish peroxidase labeled polymer (DAKO Envision System, DAKO Corporation). Immunohistochemical reactions were developed with diaminobenzidine (DAB) as chromogen. Finally, sections were lightly counterstained with 10% Mayer hematoxylin, and mounted for observation. The results for the negative control are shown in Figure S3. The concentration of antibodies (Bad, Cyt-C and Ki67) was 1∶50 in the immunohistochemical reactions. The staining was scored as the following: negative (−), weak (+), moderate and strong (++).

### Statistical analysis

Statistical analysis was carried out using Prizm 3 from GraphPad Software (San Diego, CA). Data are presented as mean ± SD. Statistical significance for comparison between groups was determined by using Student's paired two-tailed t-test. All the results were generated from three independent experiments.

## Results

### Knockdown of AQP4 by siRNA approach induced apoptosis of LN229 cells

**Figure 1:**
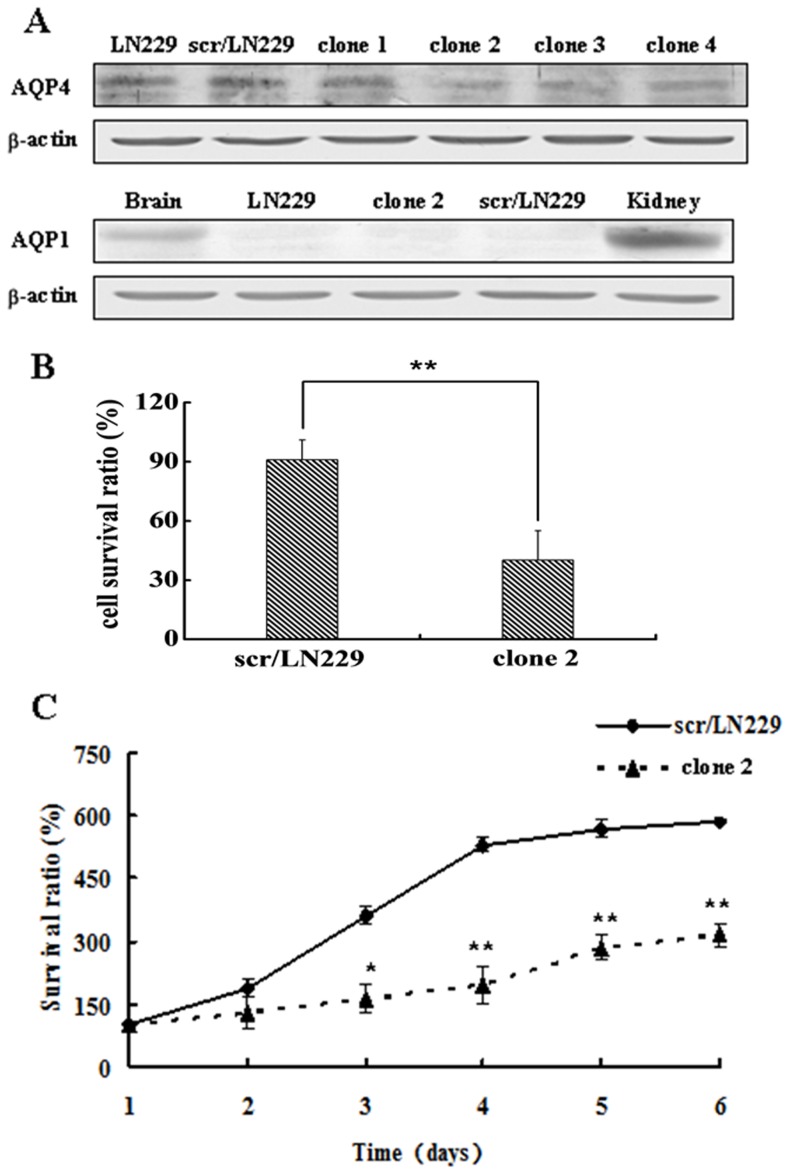
The establishment of stable LN229 cells expressing AQP4 siRNA plasmid. A. Western blotting analysis of AQP4 expression in parental LN229, scr/LN229 and siAQP4/LN229 clone 1, clone 2, clone 3 and clone 4 cells. The expression of AQP1 in parental LN229 and siAQP4/LN229 clone 2 cells were also detected, the kidney and normal brain tissue were used as positive controls, β-actin was used as a loading control. B. Cells osmotic fragility was examined after AQP4 reduction. The survival rate of the control was 71.61% ± 11.75% (mean ± SD), whereas it decreased to 29.46% ± 14.84% (mean ± SD) in siAQP4/LN229 clone 2 cells. C. Comparison of cell proliferation in scr/LN229 and siAQP4/LN229 clone 2 cells by MTT assay. Each data point was an average of triplicate assays (Bars, standard deviation; two-way ANOVA analysis, **P<0.01).

First, plasmids expressing AQP4 siRNA sequences were transfected into LN229 cells which were indicated as siAQP4/LN229 cells. A siRNA vector containing a scrambled sequence was also transfected to the LN229 cells to generate control cells appointed as scr/LN229 cells. After the G418 selection, transfected cells were screened for AQP4 expression by Western blotting. The levels of AQP4 were similar in scr/LN229 and untransfected LN229 cells. In contrast, the levels of AQP4 expressed by four clones (designated 1 to 4) derived from cells transfected with siAQP4/LN229 were 28%, 66%, 56%, and 54% of that of scr/LN229 cells. We chose siAQP4/LN229 clone 2 to perform the following functional experiments. As two aquaporins AQP1 and AQP4 expression had been reported in the brain, we also tested AQP1 expression in both LN229 and siAQP4/LN229 cells. The expression level of AQP1 was below the limit of detection of our western blotting method. In contrast, we detected high levels of expression of AQP1 in normal kidney and brain tissues (Fig. 1A).

To determine whether the reduction in AQP4 expression had functional consequences, we assessed cellular osmotic fragility. The results showed that the survival rate of the control was 71.61%±11.75% (mean ± SD), whereas it decreased to 29.46% ± 14.84% (mean ± SD) in siAQP4/LN229 clone 2 cells (Fig. 1B). The reduced osmotic fragility of siAQP4/LN229 clone 2 cells indicated that the AQP4 RNA interference in this clone was functional. Further, to study the function of AQP4 in LN229 cells, all subsequent functional assays and western blot analyses were conducted using the siAQP4/LN229 clone 2 cells.

**Figure 2:**
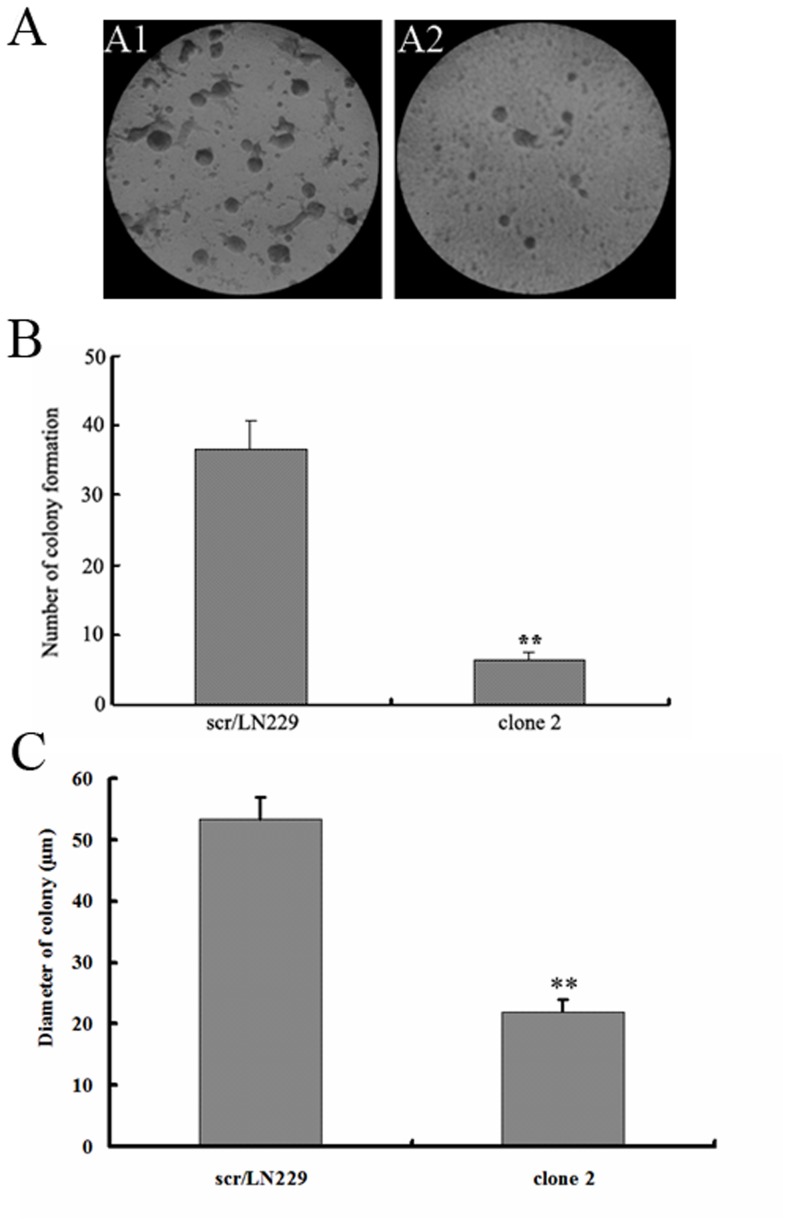
Colony formation of LN229 cells after AQP4 reduction. A. representative images of colony formation for scr/LN229 and siAQP4/LN229 clone 2 cells (200×). The images were taken at the 10th day of the assay. B. Quantitation of colony number in two groups (scr/LN2229 cells group and siAQP4/LN229 clone 2 cells group). Results were from images (100×) of three independent experiments. C. Quantitation of colony size in two groups (scr/LN2229 cells group and siAQP4/LN229 clone 2 cells group). The data was collected by representative images from three repeated experiments (200×) (two-way ANOVA analysis, **p<0.01).

MTT assay was performed to detect the cells proliferation in scr/LN229 and siAQP4/LN229 cells. The results are shown in Fig. 1C. The data was measured from the 1st day to the 6th day. The proliferation of siAQP4/LN229 clone 2 cells was greatly inhibited compared with the control cells. Colony formation assay showed that the number of colonies was significantly decreased after AQP4 reduction, and the size of colony was smaller in clone 2 cells compared with the control (Fig. 2 A, B, C).

**Figure 3:**
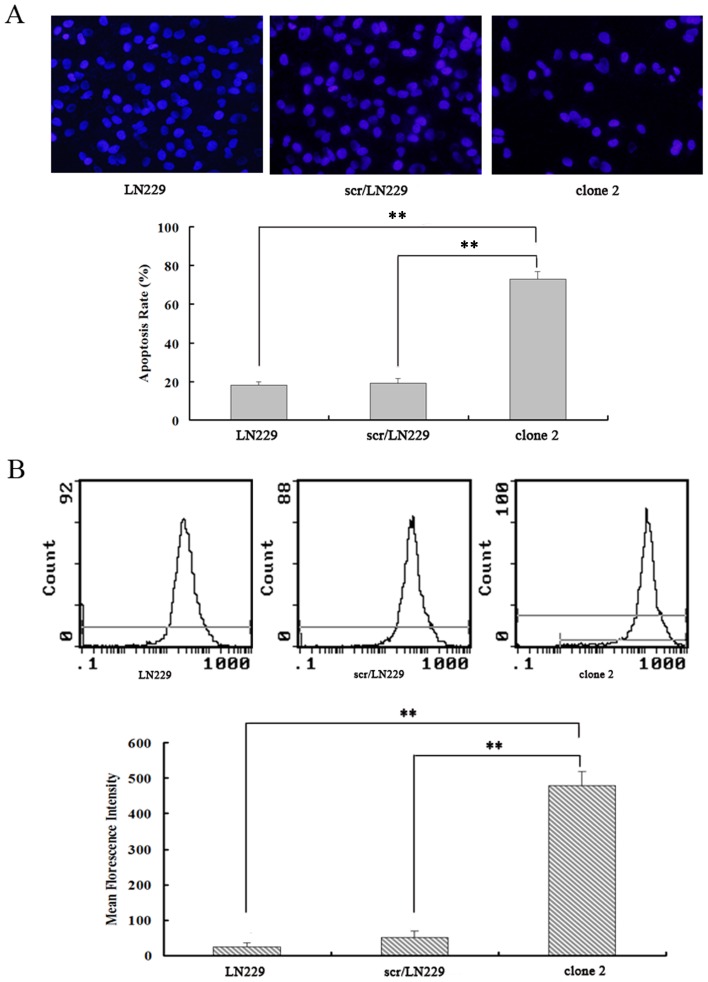
Reduction of AQP4 induced apoptosis of LN229 cells. A. Images showed DAPI staining of LN229, scr/LN229 and siAQP4/LN229 clone 2 cells (200×). LN229 and scr/LN229 groups were regarded as control. The quantitative result was shown by the histograms (**P<0.01). Quantitative results were analyzed (two-way ANOVA analysis, **P<0.01). B. In order to detect the Cyt-C efflux from mitochondria, the cells were incubated with anti-Cyt-C antibody at room temperature for 60 min in the dark, and then the content of Cyt-C was detected by flow cytometry. The quantitative result was shown by the histograms (**P<0.01).

**Figure 4:**
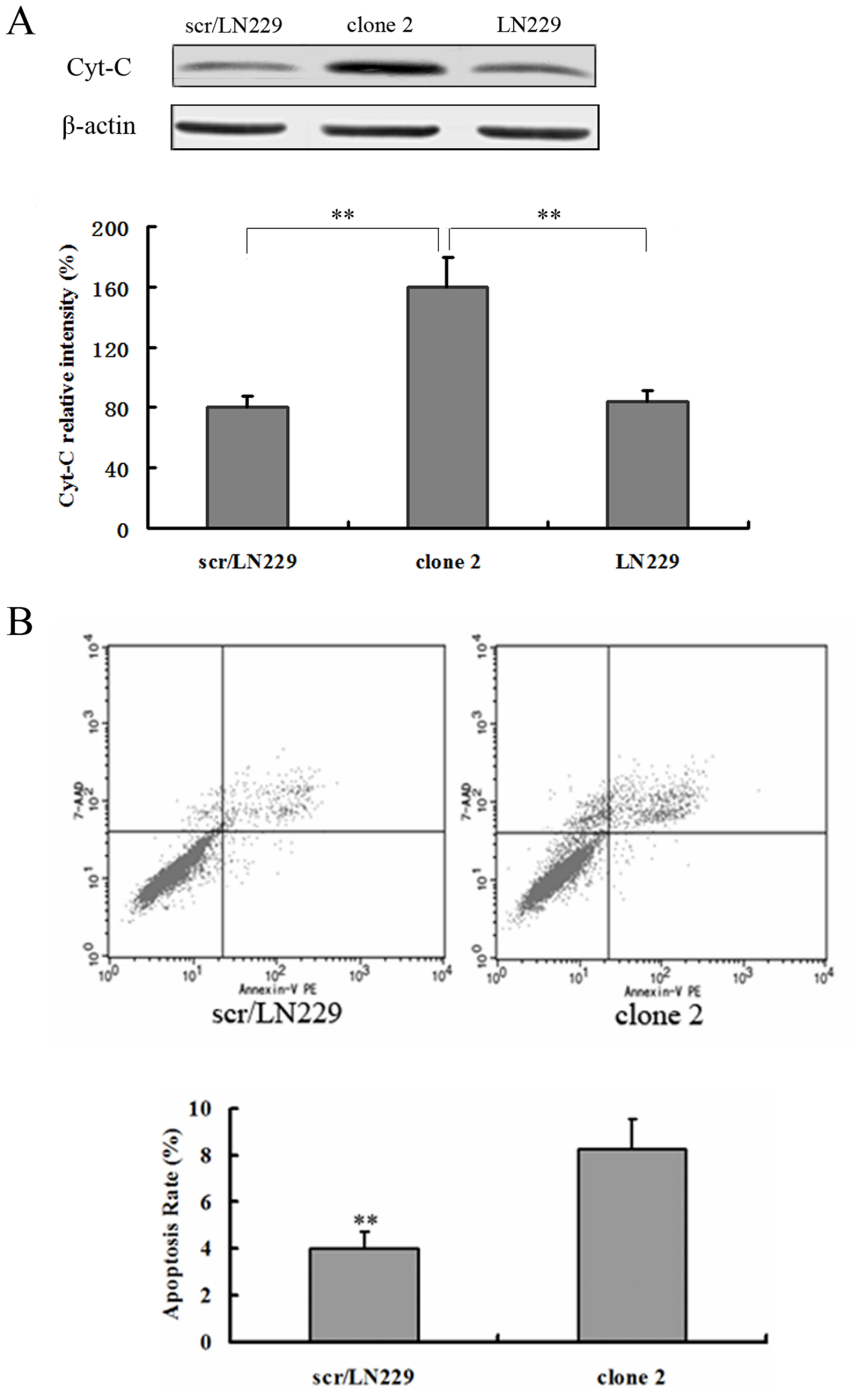
Reduction of AQP4 induced apoptosis of LN229 cells. A. Protein level of Cyt-C was determined by Western blotting. Expression of Cyt-C was quantitated by densitometry and normalized to β-actin expression. Results were analyzed using two-way ANOVA analysis, **P<0.01. Western blotting results showed a representative blot taken from three independent experiments. B. Annexin V Apoptosis Kit was used to detect cells apoptosis. Flow cytometric analyses of scr/LN229 and siAQP4/LN229 clone 2 cells were shown. Apoptosis rate in scr/LN229 cells and in siAQP4/LN229 clone 2 cells were (3.8±0.71)% and (8.28±1.25)%, respectively.

We next investigated the effect of inhibiting the expression of AQP4 on glioma cell apoptosis. DAPI staining was performed to detect changes in the morphology of nuclei in apoptotic cells. We used DAPI staining to detect the apoptotic cells after AQP4 reduction in LN229 cells. As shown in Fig. 3A, the apoptosis rate in scr/LN229 was 17.89%±2.32%, whereas it was 72.56%±3.4% in siAQP4/LN229 clone 2 cells. LN229 cells showed the similar apoptosis rate to the scr/LN229 cells. It was well known that the efflux of Cytochrome C from mitochondria is the initial step leading to cell apoptosis [15]. Therefore, we detected the cytoplasmic Cytochrome C levels by flow cytometry. siAQP4/LN229 clone 2 cells showed higher levels of Cytochrome C compared with the scr/LN229 cells and LN229 cells (Fig. 3B). We also performed western blotting analysis of cytoplasmic cytochrome C expression and found that siAQP4/LN229 clone 2 cells expressed higher levels of cytochrome C than those of scr/LN229 and LN229 cells (Fig. 4A), consistent with the results of flow cytometry. Next, we determined the extent of apoptosis using a kit for detecting annexin V and found that a higher percentage of siAQP4/LN229 clone 2 cells were apoptotic compared with scr/LN229 control cells (Fig. 4B).

**Figure 5:**
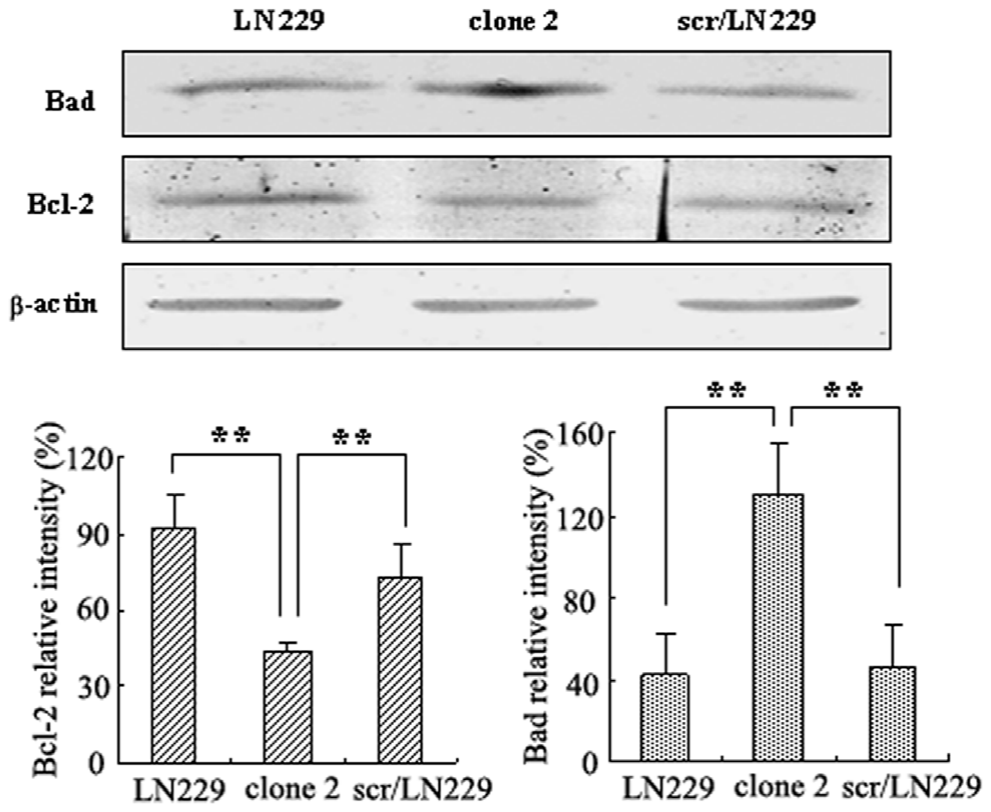
The expression of Bad and Bcl-2 in LN229 cells were detected by Western blotting after AQP4 reduction. Western blotting was performed by using either anti-Bad antibody or anti-Bcl-2 antibody. Expression of Bad and Bcl-2 were quantitated by densitometry and normalized to β-actin expression. Data were analyzed using two-way ANOVA analysis **P<0.01.

Bad and Bcl-2 are pro-apoptotic and anti-apoptotic members, respectively, in the Bcl-2 family. Bad induces apoptosis by forming heterodimers with Bcl-2, which inhibit the anti-apoptotic activity of Bcl-2 [16]. In the present study, Bcl-2 and Bad protein expression were studied upon AQP4 reduction by Western blotting. We found that protein level of Bad was dramatically increased in siAQP4/LN229 clone 2 cells, while the level of Bcl-2 was significantly decreased compared with those in the scr/LN229cells (Fig. 5, P<0.01).

### Inhibitor of AQP4, PMA, led to LN229 cells apoptosis

**Figure 6:**
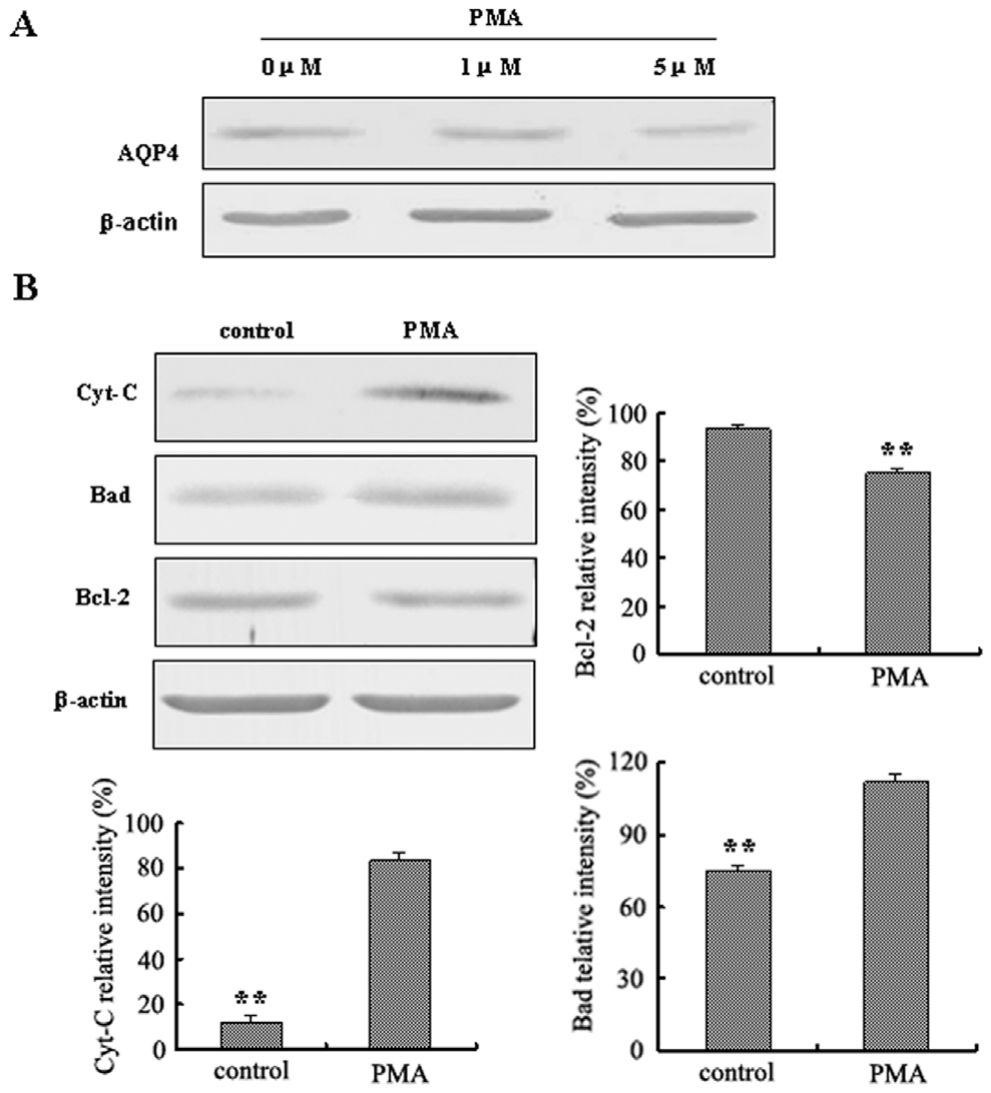
Reduction of AQP4 by PMA in LN229 cells induced apoptosis. A. The expression of AQP4 in LN229 cells with PMA (0 µM, 1 µM, 5 µM) treatment (24 hr) was detected by Western blotting. B. Western blotting was performed by using anti-Cyt-C, anti-Bad or anti-Bcl-2 antibody. (5 µM of PMA was used.) Expression of Cyt-C, Bad and Bcl-2 were quantitated by densitometry and normalized to β-actin expression. Data were analyzed using two-way ANOVA analysis **P<0.01.

Besides of applying AQP4 RNA interference for reduction of AQP4 protein levels, we also used the AQP4 inhibitor phorbol 12-myristate 13-acetate (PMA) which can activate protein kinase C (PKC) and inhibit AQP4 expression [17]. Western blotting results showed that the expression of AQP4 was significantly reduced with PMA (1 µM, 5 µM) treatment compared with control (Fig. 6A). We chose 5 µM as a final concentration to detect the expression of Cytochrome C, Bad and Bcl-2 by Western Blotting. Densitometric analysis of Western blotting from triplicate samples was performed. The expression of Cytochrome C and Bad were increased after treatment with PMA (5 µM). The relative intensity of Cytochrome C increased 6.7 fold compared with that of control and the relative intensity of Bad increased 1.5 fold compared with that of control. In contrast, Bcl-2 expression was decreased compared with the control (Fig. 6B, P<0.01). The data are consistent with the results of the RNA interference experiments.

### Knockdown of AQP4 expression by siRNA induces apoptosis of U87 cells

**Figure 7:**
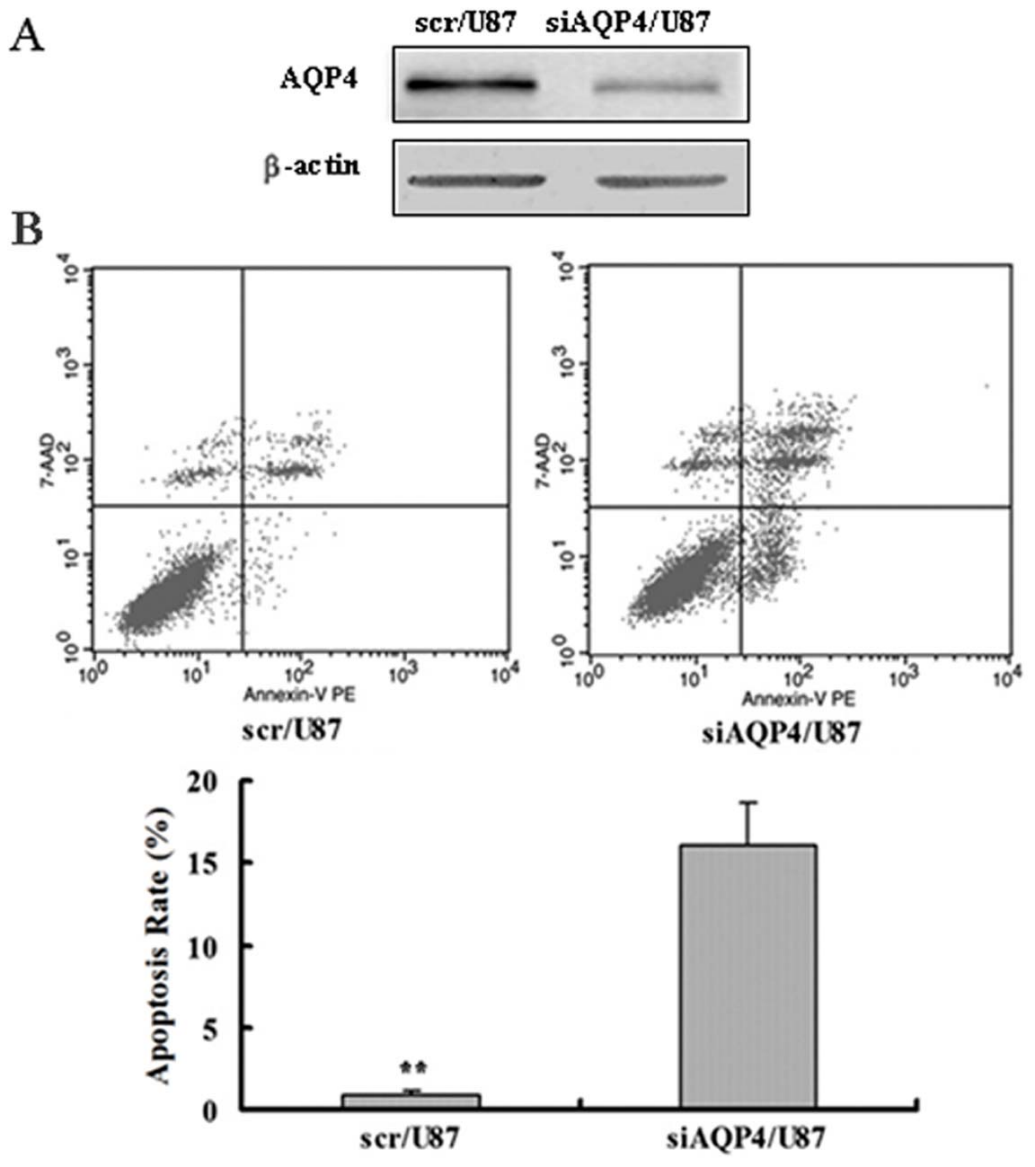
Annexin V Apoptosis Kit was used to detect cells apoptosis of U87 cells with transient transfection of AQP4 siRNA. A. Western blotting analysis of AQP4 expression in scr/U87 and siAQP4/U87 cells. B. Flow cytometric analysis of U87 cells with transient transfection of AQP4 siRNA by Annexin V Apoptosis Kit. Apoptosis rate in scr/U87 cells was (0.81±0.11)% and it was (16.07±2.43)% in siAQP4/U87 cells. Data was representative of three independent experiments. Data were analyzed using two-way ANOVA analysis **P<0.01.

We also used another glioblastoma cell line U87 to confirm the function of AQP4 in glioblastoma cell apoptosis. First, AQP4 siRNA was transiently transfected into U87 cells, the western blotting result showed that the expression of AQP4 was reduced significantly in siAQP4/U87 cells compared with the control scr/U87 cells (Fig. 7A). Then, Annexin V Apoptosis Kit was applied by flow cytometry to confirm the role of AQP4 in U87 cells apoptosis. As shown in Fig. 7B, the percentage of apoptotic cells in the control group was 0.81%±0.11%, whereas siAQP4/U87 cells had apoptotic rate of 16.07%±2.43%, the quantitative results showed a significant difference between scr/U87 and siAQP4/U87 cells. These results suggested that reduction of AQP4 also induced apoptosis of U87 cells, which is consistent with the results for LN229 cells.

### Reduction of AQP4 induced apoptosis in a mouse xenograft model

**Figure 8:**
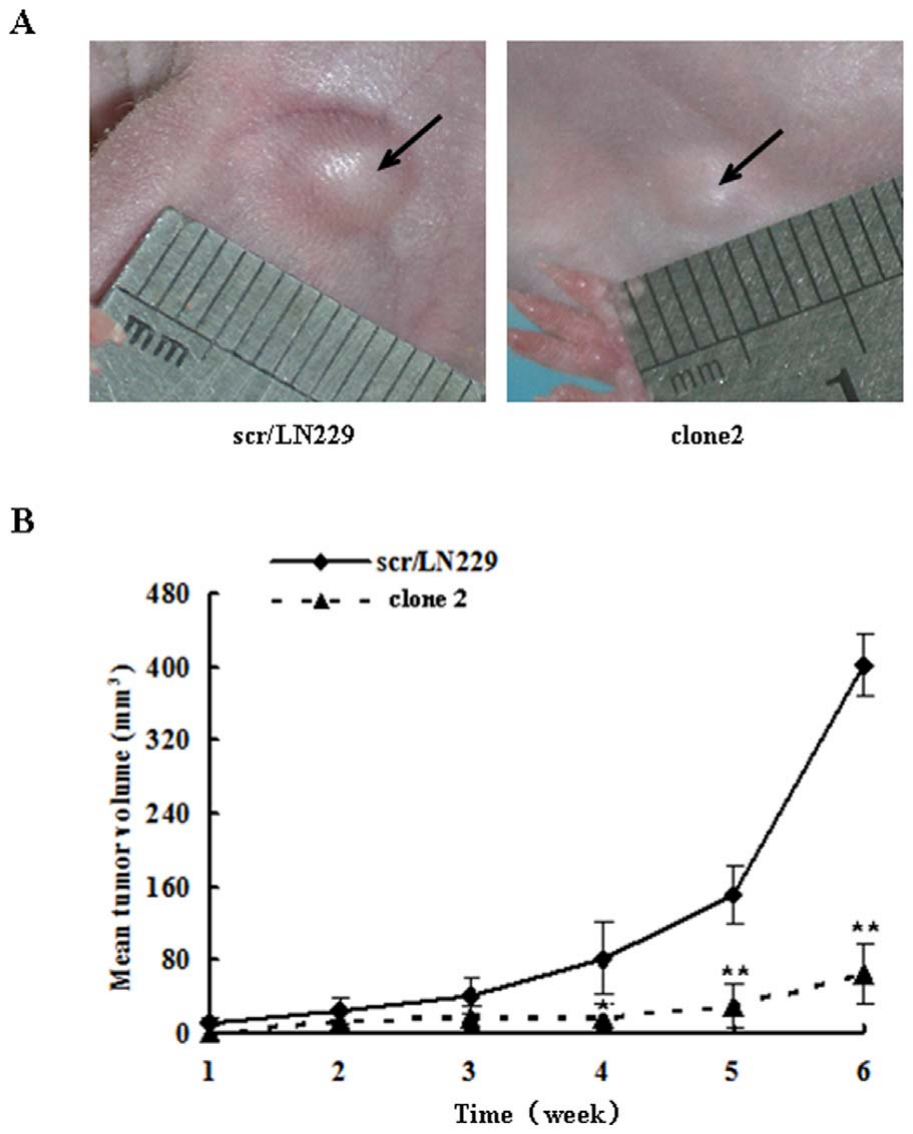
Reduction of AQP4 induced glioma cells apoptosis in *in vivo* assay. (A) Stable clones of scr/LN229 and siAQP4/LN229 were subcutaneous injected into Nu/Nu mice respectively. The size of tumors was measured each week. 6 weeks later, the representative images of tumor size in each group were captured. (B) The results of tumor volume *in vivo* were analyzed by two-way ANOVA analysis, **P<0.01.

To determine if the *in vitro* assays described above have any bearing on tumorigenicity *in vivo*, we applied the subcutaneous mouse xenograft model to validate the role of AQP4 in glioblastoma apoptosis *in vivo*. We used 48 Nu/Nu mice which were divided into 2 groups, and each group was injected with siAQP4/LN229 clone 2 cells or scr/LN229 clone cells respectively. The size of tumors was measured each week. Six weeks later, mice were sacrificed. After the mice were sacrificed, the tumors were dissected and the size was measured by a digital caliper. Tumor growth in the siAQP4/LN229 clone 2 group mice was significantly slower than that in the control group mice (Fig. 8A). The endpoint volume of control group was 402±34 mm^3^ compared with 65 ± 32 mm^3^ for the siAQP4/LN229 clone 2 group. The tumor volume of the two groups began to diverge since the 4th week (P<0.05), and the divergence has become much significant in the 5th week (P<0.01). The tumor volume of siAQP4/LN229 clone 2 group showed great reduction compared with the control mice (Fig. 8B).

**Figure 9:**
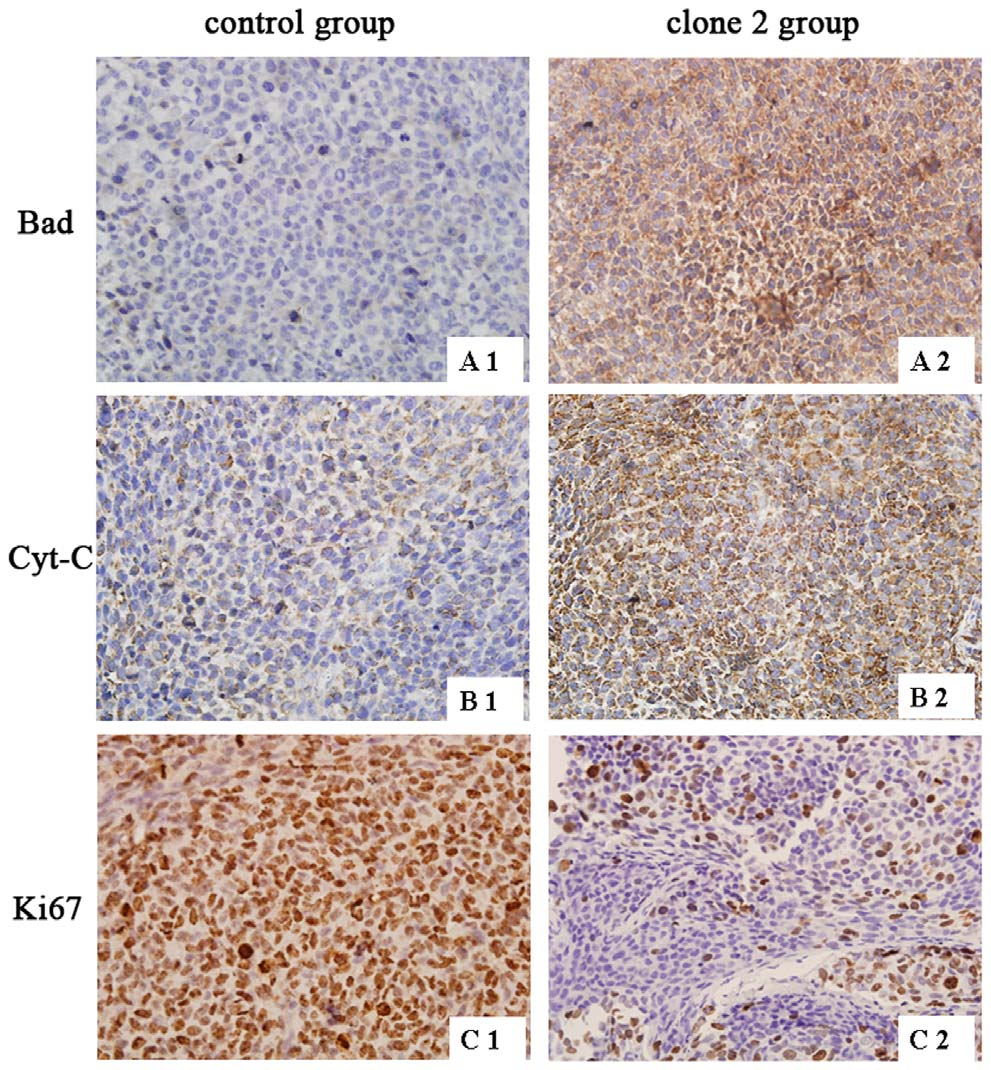
The expression of Bad, Cyt-C and Ki67 in xenograft tumor tissues. Xenograft tumor tissues were obtained from subcutaneously inoculated Nu/Nu mice which were sectioned for immunohistochemistry to detect the expression of Bad, Cyt-C and Ki67. A1, B1 and C1 were images of scr/LN229 group, A2, B2 and C2 were images of siAQP4/LN229 clone 2 group (400×).

We also applied transplantation tumor specimens to detect the expression of Cytochrome C, Bad and Ki67 by immunohistochemisty. The result showed that the expression of Cytochrome C and Bad were increased in the siAQP4/LN229 clone 2 group, which was consistent with the result of Western blotting *in vitro*. In contrast, the Ki67 expression was decreased in siAQP4/LN229 clone 2 group, which revealed a low cell proliferation compared with the control group (Fig. 9). Quantification of the immunohistochemical analysis is shown in Table 1. Statistical analysis was used two independent samples tests.

**Table 1:** The expression of Bad,Cyt-C and Ki67 in xenografts.

Group	Cases	−	+	++	*Z*	*P value*
**Bad**						
**clone 2**	24	0	7	17	−1.975	0.048
**control 24**	24	18	6	0		
**Cyt-C**						
**clone 2**	24	1	4	19	−2.152	0.031
**control 24**	24	12	9	3		
**Ki67**						
**clone 2**	24	13	7	4	−2.303	0.021
**control 24**	24	2	5	17		

## Discussion

Apoptosis is a highly sophisticated and elaborate mode of cell death that requires precise regulation of different intracellular signaling pathways to ensure the continuation of the transmission of the death signal [11]. Previous studies have shown that potassium efflux or a decrease in intracellular K^+^ concentration appears to occur early in the cell death program and may regulate a number of apoptotic events, including caspase and nuclease activation. [18], [19]. The AVD is one of the earliest and most conserved morphological events in apoptosis. AQPs play a role in the movement of water across the plasma membrane in dying cells during AVD. Inactivation of AQPs after AVD could help to maintain the low K^+^ concentration which is essential in apoptotic cells [8]. These studies suggested that the AQPs play important roles in cell apoptosis.

In the present study, we demonstrate that AQP4 directly participated in glioblastoma cell apoptosis *in vitro* and *in vivo*. Thus, when we treated U87 cells with PMA, which can inhibit AQP4 expression, apoptosis was induced. PMA, as a PKC activator, also has a spectrum of other effects. Our findings showing that AQP4 expression was inhibited with an AQP4-specific siRNA and led to apoptosis confirmed the results of the PMA experiments. We also found that inhibiting AQP4 expression resulted in increased expression of Bad and decreased expression of Bcl-2. This may represent a possible mechanism for glioblastoma cell apoptosis, because Bad promotes apoptosis while Bcl-2 exerts the opposite effect.

A pivotal event in the intrinsic pathway of apoptosis is the release of cytochrome C from the mitochondrial intermembrane space [20]. Mitochondrial cytochrome C release occurs via volume-dependent mechanisms, which are based on the swelling of mitochondria, leading to permeabilization of the outer mitochondrial membrane [21]. Recently, a novel mechanism for osmotic swelling of mitochondria has been described. AQP8 and AQP9 channels are present in the inner mitochondrial membranes of various tissues, including the kidney, liver and brain where they may mediate water transport associated with physiological volume changes, which contribute to the osmotic swelling induced by apoptotic stimuli [22]. Our present study shows that the level of cytochrome C was increased after AQP4 expression was reduced. These results indicate that AQP4 acts as a critical factor in the regulation of glioblastoma cell apoptosis may through mitochondrial survival signaling. However, further investigation is required to unravel the signaling pathway leading from the reduction of AQP4 expression to the initiation of apoptosis as indicated by the changes in expression and activities of the key apoptotic molecules.

The results of our animal experiments also support the role of AQP4 in the glioblastoma cells apoptosis. We used the subcutaneous model in the present study and showed that the tumor volume of control group was 402±34 mm^3^ and the siAQP4/LN229 clone 2 group was 65±32 mm^3^ at the end time point. The volumes of tumors of experimental group were significantly reduced compared with those of the controls. Although the subcutaneous xenograft model has been widely used to study tumors, an intracranial transplantation model may provide better survival data for glioblastoma and should be used in future research.

Although the role of AQP in apoptosis is indicated by its participation in AVD, the role of AQP4 in glioblastoma apoptosis remains to be elucidated. In the present study, we provide evidence that AQP4 acts as a critical factor in the regulation of apoptosis may through mitochondrial survival signaling. Moreover, AQP4 may serve as a new anti-apoptosis target for therapy of glioblastoma.

## Supporting Information

Figure S1 **Original western blot results.** Molecular standards are shown. The order of the western blot results in these supplemental Figures corresponds to their order in the manuscript.
(TIF)

Figure S2 **Original western blot results.** Molecular standards are shown. The order of the western blot results in these supplemental Figures corresponds to their order in the manuscript.
(TIF)

Figure S3 **Images of negative control (non-specific antibody was used) for immunohistochemistry staining were shown.** A was image of scr/LN229 control group; B was image of siAQP4/LN229 clone 2 group (400×).
(TIF)
