# Supplementary figures and images for: Knockdown a Water Channel Protein, Aquaporin-4, Induced Glioblastoma Cell Apoptosis
Source: PLoS One. 2013 Aug 12;8(8):e66751. doi: 10.1371/journal.pone.0066751 (PMC3741385; doi:10.1371/journal.pone.0066751)

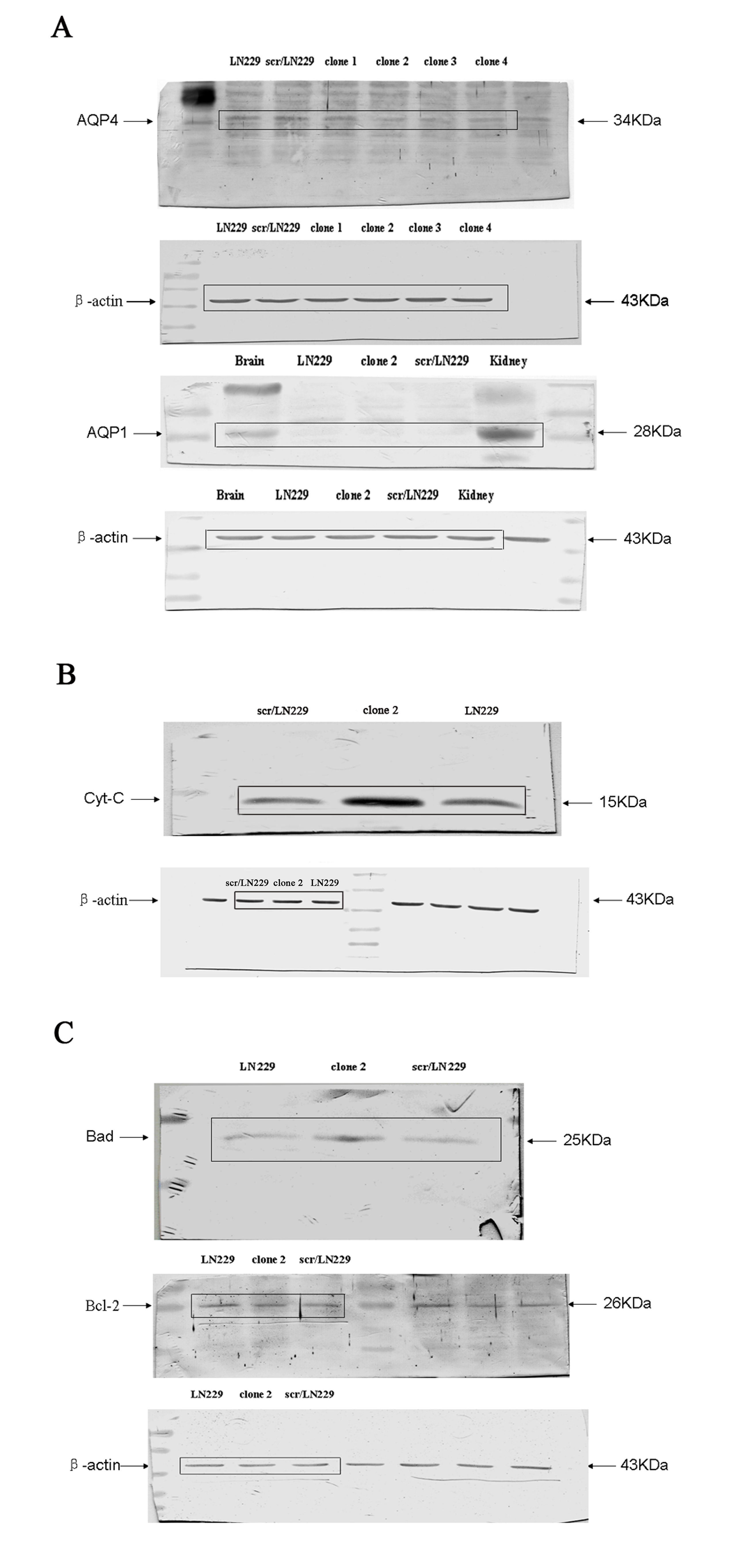

Supplement: Figure S1 — Original western blot results. Molecular standards are shown. The order of the western blot results in these supplemental Figures corresponds to their order in the manuscript. (TIF) [file pone.0066751.s001.tif]

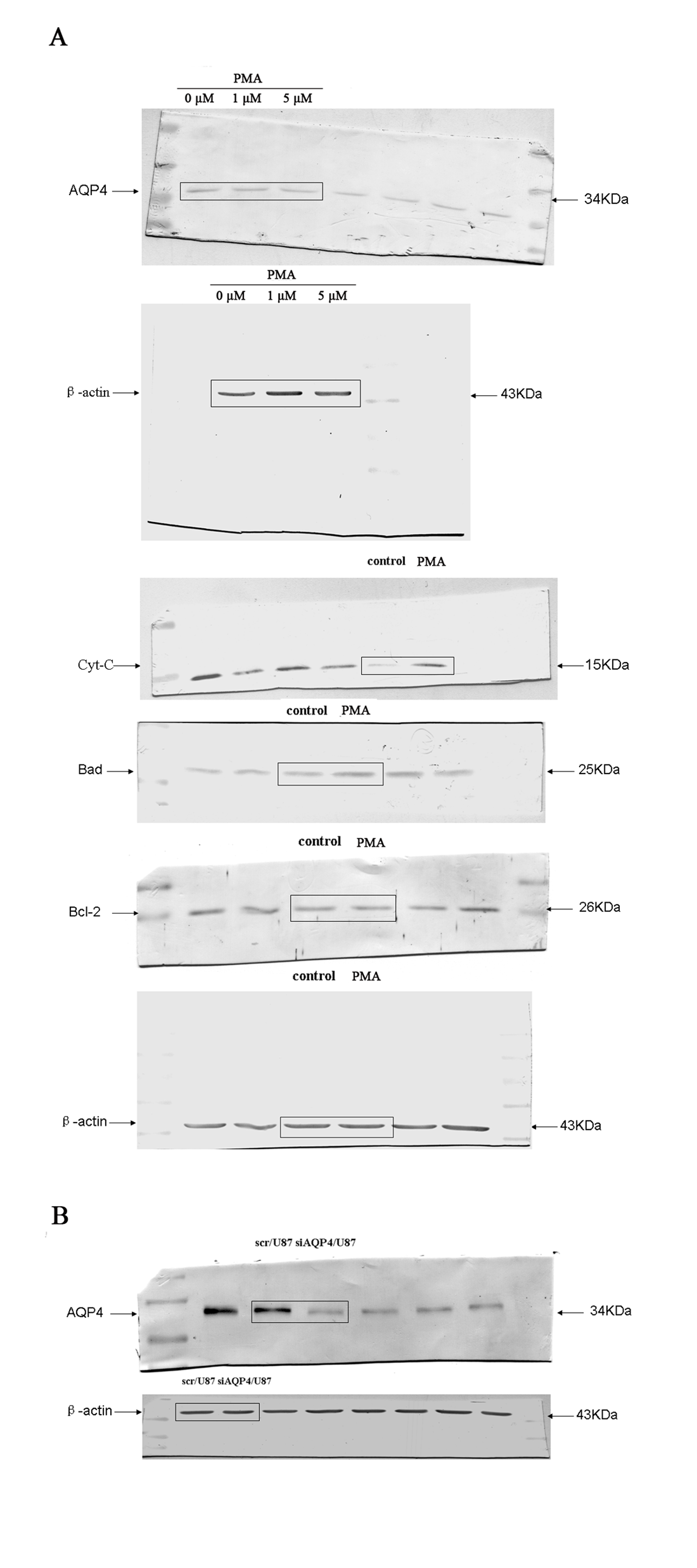

Supplement: Figure S2 — Original western blot results. Molecular standards are shown. The order of the western blot results in these supplemental Figures corresponds to their order in the manuscript. (TIF) [file pone.0066751.s002.tif]

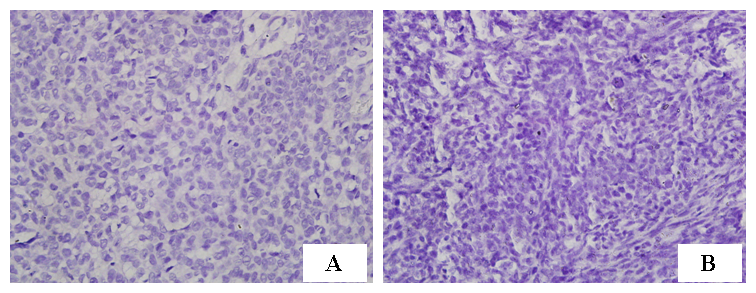

Supplement: Figure S3 — Images of negative control (non-specific antibody was used) for immunohistochemistry staining were shown. A was image of scr/LN229 control group; B was image of siAQP4/LN229 clone 2 group (400×). (TIF) [file pone.0066751.s003.tif]
